# Validation and assessment of psychometric properties of the Greek Eating Behaviors Assessment for Obesity (GR-EBA-O)

**DOI:** 10.1007/s40519-024-01664-6

**Published:** 2024-05-11

**Authors:** Panagiota Mavrandrea, Matteo Aloi, Matteo Geraci, Androula Savva, Fragiskos Gonidakis, Cristina Segura-Garcia

**Affiliations:** 1 Eating Disorders Unit, 1st Department of Psychiatry, National and Kapodistrian University of Athens, Eginition Hospital, Vas Sofias 74, 11526 Athens, Greece; 2 Department of Clinical and Experimental Medicine, University of Messina, Messina, Italy; 3 Department of Health Sciences, University “Magna Graecia” of Catanzaro, Catanzaro, Italy; 4 Department of Clinical Neuroscience, Psychology Division, Karolinska Institutet, Stockholm, Sweden; 5 Department of Medical and Surgical Sciences, University “Magna Graecia” of Catanzaro, Catanzaro, Italy; 6 Center for Clinical Research and Treatment of Eating Disorders, University Hospital Mater Domini, Catanzaro, Italy

**Keywords:** Obesity, Eating behavior, Assessment, Greek validation, Psychometric properties

## Abstract

**Introduction:**

With increasing morbidity and risk of death, obesity has become a serious health problem largely attributable to difficulties in finding proper treatments for related diseases. Many studies show how detecting abnormal eating behaviors could be useful in developing effective clinical treatments. This study aims at validating the Greek version of the Eating Behaviors Assessment for Obesity (EBA-O).

**Method:**

After a double English/Greek forward/backward translation of the EBA-O, 294 participants completed the Greek version (GR-EBA-O), the Eating Disorder Examination Questionnaire, the Binge Eating Scale, and the Yale Food Addiction Scale. Confirmatory factor analysis (CFA) and construct validity were calculated, and Two-way MANOVA was computed with the factors of GR-EBA-O controlling for sex and BMI categories.

**Results:**

CFA confirmed the second-order five factors (i.e., food addiction, night eating, binge eating, sweet eating, and prandial hyperphagia) structure of the original EBA-O with excellent fit indices. GR-EBA-O factors were highly correlated. The GR-EBA-O subscales were also significantly correlated with the remaining measures, demonstrating good concurrent validity.

**Conclusion:**

The Greek version of the EBA-O has demonstrated sound psychometric properties and appears a reliable and user-friendly tool to identify pathological eating behaviors in obesity.

*Level of evidence*: V, descriptive research.

## Introduction

Obesity is a complex and multifactorial condition that not only represents a significant health problem with an increasing risk of mortality [1] but is also frequently associated with comorbidities [2, 3] and high rates of treatment dropout among patients [4].

Increasing evidence indicates that binge eating disorder (BED) impacts a subpopulation of patients dealing with obesity [5]. In fact, individuals with BED are 3–6 times more likely to be obese compared to those without an eating disorder [6, 7]. BED is also linked to an early onset of increased weight and obesity, with around 30% of individuals with binge eating behaviors reporting a history of childhood obesity [8]. Additionally, family negative influences related to shape, weight, and eating, along with concerns about body shape and weight, have all been associated with a diagnosis of BED [9]. Binge eating is more prevalent among those pursuing weight loss approaches, with a percentage ranging from 9 to 29% reporting episodes of binge eating [10].

Clinical practice has revealed that people facing obesity display distinctive pathological eating habits that may need different therapeutic approaches [11]. The need for tailored therapeutic interventions has made the phenotyping of obesity essential [12, 13]. Research indicates that pathological eating behaviors can play a role in phenotyping individuals with obesity [14].

These behaviors can include binge eating, where a person consumes a large amount of food in a short time and feels like they can't control their eating [15]. Night eating is also common among those with night eating syndrome (NES), a condition recognized in the DSM-5 as a type of eating disorder. NES is characterized by two primary indicators: evening hyperphagia and/or eating during the night. Diagnosis requires identifying these behaviors along with at least three out of five key symptoms: morning anorexia, post-dinner eating, insomnia, depression, and the belief that eating is necessary for sleep [16]. Food addiction is the compulsive consumption of high-fat and high-sugar processed foods, and it is closely associated with Binge Eating Disorder (BED) and obesity [17, 18]. Conversely, indulging in sweet foods or beverages as a response to emotional triggers is referred to as sweet eating [19]. Finally, hyperphagia denotes an elevated or disproportionate hunger leading to the consumption of substantial amounts of food, often surpassing the body's nutritional requirements, particularly during main meals; people experiencing hyperphagia commonly consume larger portions of food compared to others or opt for additional servings.

Recently, a questionnaire has been validated, demonstrating effectiveness in identifying and determining the severity of pathological eating behaviors typical of patients with obesity: the Eating Behavior Assessment for Obesity (EBA-O) [20]. The EBA-O is an 18-item questionnaire that evaluates the presence and severity, in the last three months, of five pathological eating behaviors typically observed in patients with obesity: night eating, food addiction, sweet eating, hyperphagia, and binge eating. Therefore, its main characteristic is that it can simultaneously evaluate various dysfunctional eating behaviors in patients with obesity. While there are numerous tools in the literature that assess these behaviors separately, using them would require more time for both administration and scoring [21–23]. According to its authors, the primary purpose of the EBA-O is to provide clinicians and researchers an easy tool to administer and score that can be used by health care providers without expertise in the field of EDs [20] in order to evaluate pathological eating behaviors frequently associated with obesity.

Based on the above, the aim of the present study is to validate and assess the psychometric properties of EBA-O questionnaire (i.e. factor structure, internal consistency, construct validity) in a sample of patients with obesity in the Greek population.

## Methods

### Participants

A convenience sample was recruited from the general population between October 2022 and March 2023 through an online survey. Participants were informed about the study's purpose, procedures, the voluntary nature of participation, anonymity, and data management and storage. They provided consent by clicking on the consent box. Socio-demographic data, including age, gender, education, occupation, height, weight, and lifestyle, were collected.

Inclusion criteria comprised men and women, aged between 18 and 65 years, with a BMI ≥ 25 kg/m^2^. A total of 294 individuals, all of Greek nationality, participated by clicking on the consent box, with 260 of them being women (88%). All participants completed the battery of measures. Responses obtained  from individuals younger than 18 or older than 65 years and from those with BMI < 25 kg/m^2^ were excluded from the analysis.

### Measures

#### Eating Behaviors Assessment for Obesity (EBA-O)

The authors made a double English/Greek forward/backward translation of the EBA-O as follows: once an initial agreement was reached among translators from English to Greek, another researcher, blind to this original version, made the translation back into English. After verifying the similarity with the original test, the newly developed GR-EBA-O was given to a small group of 15 volunteers who evaluated the comprehensibility of the items. All raters considered it to be clear and easy to rate.

The GR-EBA-O consists of 18 item rated with an 8-point Likert type scale ranging from 0 (never) to 7 (everyday) in order to assess the presence and severity, in the last three months, of five pathological eating behaviors representative of obesity: night eating, food addiction, sweet eating, hyperphagia, and binge eating (Appendix 1).

To assess the convergent validity of the EBA-O, participants were asked to complete the following tests:

- Binge Eating Scale (BES) [24]: A 16-item test measuring the severity of binge eating; scores < 17, 17–27, and > 27 indicate that the risk of an individual suffering from BED is unlikely, possible, and probable, respectively. The internal consistency in this study was McDonald’s ω = 0.88.
- Yale Food Addiction Scale (YFAS) [25]: This scale assesses addiction-like eating behavior in the past 12 months through 25 items, scored on an eight-point scale ranging from never (score = 0) to every day (score = 7), representing 11 symptoms. The Kuder–Richardson coefficient of reliability as internal consistency for the YFAS in this study was 0.83.
- Eating Disorder Examination-Questionnaire (EDE-Q) [26]: it is a self-administered questionnaire with 28 questions investigating eating psychopathology in the last four weeks. It allows obtaining scores related to four subscales (restraint, eating concern, weight concern, and shape concern) and a total score. In the present study, we found the following McDonald’s ω internal consistency reliability indexes: Restraint = 0.75; Eating Concern = 0.77; Weight Concern = 0.78; Shape Concern = 0.83; Global score = 0.84.

### Statistical analysis

A second-order five factor model, trough confirmatory factor analysis (CFA), was run using the open-source JASP software (JASP, version 0.16.4, University of Amsterdam). This aimed to assess the underlying factor structure of the EBA-O and validate the suitability of a total score. The choice to employ the diagonally weighted least squares (DWLS) estimator, utilizing a polychoric correlation matrix, was made to effectively estimate the parameters, as it stands out as the most suitable method for modeling ordered data. The assessment of model fit utilized several indices: the relative chi-square (χ^2^/df), Tucker-Lewis index (TLI), comparative fit index (CFI), root mean square error of approximation (RMSEA), and standardized root mean square residual (SRMR). Adequate values were considered as follows: TLI and CFI ≥ 0.90 (adequate) and ≥ 0.95 (very good), RMSEA ≤ 0.08 (adequate) and ≤ 0.05 (very good), and an SRMR close to 0.08. Additionally, good fit was indicated by χ^2^/df values < 3.0 and very good fit by values < 2.0, aligning with the guidelines proposed by Hu and Bentler [27]. To establish construct validity, correlations between the EBA-O factors and respective questionnaires were examined, emphasizing correlation coefficients (r) greater than 0.30 as recommended benchmarks.

Two-way multivariate analysis of variance (two-way MANOVA) was carried out with the five factors of EBA-O as independent variables, and sex and categorical BMI as dependent variables. Eta-squared (η^2^) was used as a measure of the effect size of MANOVA considering values of 0.01, 0.06, and 0.14 as indicating small, medium, and large effects, respectively. The Bonferroni correction was used to correct for multiple comparisons (p = 0.05/10 = 0.005).

## Results

Out of the initial 294 participants from whom responses were gathered, 71 were excluded due to having a BMI < 25. Characteristics of the 223 participants are detailed in Table 1.

**Table 1:** Socio demographic features of the sample

		Fr	%
Sex	F	192	86.1
	M	31	13.9
Age		35.2	9.1
BMI		31.3	5.8
BMI category	25–30	121	54.3
	30–35	53	23.8
	35–40	24	10.8
	> 40	25	11.2
Education	High school	130	58.3
	University	23	10.3
	PhD/specialization	68	30.5
	Other	2	0.9
Occupation	Housewife	12	5.4
	Not working	20	9
	Working	166	74.4
	Retired	3	1.3
	Student	20	9
	Other	2	0.9
Comorbidity	0	143	64.4
	1	79	35.6
Diets	0	17	7.6
	1–2	50	22.4
	3–4	49	22
	> 5	107	48

### Confirmatory factor analysis

**Fig. 1:**
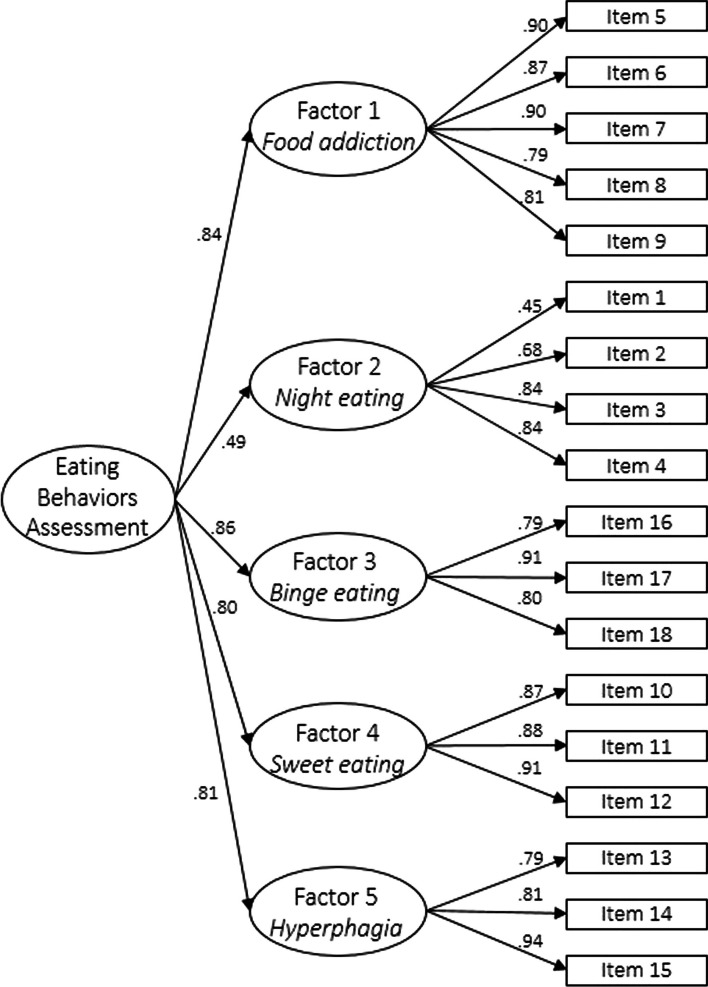
Path diagram of the second-order five-factor model of the GR-EBA-O with reported standardized coefficients of first- and second-order loadings. All values are significant for p < 0.001

The CFA exhibited excellent fit indices: CFI = 0.99, TLI = 0.99, RMSEA = 0.03, relative chi-square (χ^2^/df) = 1.21, p = 0.06. These results suggest alignment with the tested second-order five factor GR-EBA-O model (Fig. 1).

### Internal consistency (McDonald’s ω)

The internal consistency for the total score, evaluated by McDonald’s ω coefficient, was very high (ω = 0.94), indicating the excellent reliability of the GR-EBA-O.

Regarding the McDonald’s coefficient ω of all factors, it was very high indicating very good reliability: food addiction = 0.92; night eating = 0.82; binge eating = 0.87; sweet eating = 0.91; and hyperphagia = 0.89.

The factors were highly correlated with each other. The highest correlation was between factors 3 and 5 and the lowest between factors 2 and 4 (Table 2).

**Table 2:** Correlations between factors of the GR-EBA-O

	Factor 1	Factor 2	Factor 3	Factor 4	Factor 5
Factor 1 Food addiction	–				
Factor 2 Night eating	0.389^**^	–			
Factor 3 Binge eating	0.615^**^	0.340^**^	–		
Factor 4 Sweet eating	0.709^**^	0.309^**^	0.506^**^	–	
Factor 5 Hyperphagia	0.557^**^	0.372^**^	0.782^**^	0.500^**^	–

^**^p < 0.001

### Concurrent validity

Correlation analysis (Table 3) demonstrates notably significant correlations between the GR-EBA-O subscales and the BES (from 0.355 to 0.533), YFAS (from 0.294 to 0.451), and EDE-Q total score (from 0.212 to 0.616).

**Table 3:** Results of convergent validity

	Factor 1 Food addiction	Factor 2 Night eating	Factor 3 Binge eating	Factor 4 Sweet eating	Factor 5 Hyperphagia
BES	0.533^**^	0.355^**^	0.519^**^	0.410^**^	0.455^**^
YFAS symptom count	0.434^**^	0.332^**^	0.451^**^	0.294^**^	0.430^**^
EDE-Q Total score	0.562^**^	0.212^**^	0.616^**^	0.430^**^	0.471^**^

*BES* Binge Eating Scale, *YFAS* Yale Food Addiction Scale

**p < 0.001

### Two-way MANOVA

There were non-significant differences in GR-EBA-O subscales based on sex (F = 1.127, p = 0.347; Wilk’s lambda = 0.974), categorical BMI (F = 1.103, p = 0.349; Wilk’s lambda = 0.926) and their interaction (F = 1.477, p = 0.145; Wilk’s lambda = 0.934).

**Fig. 2:**
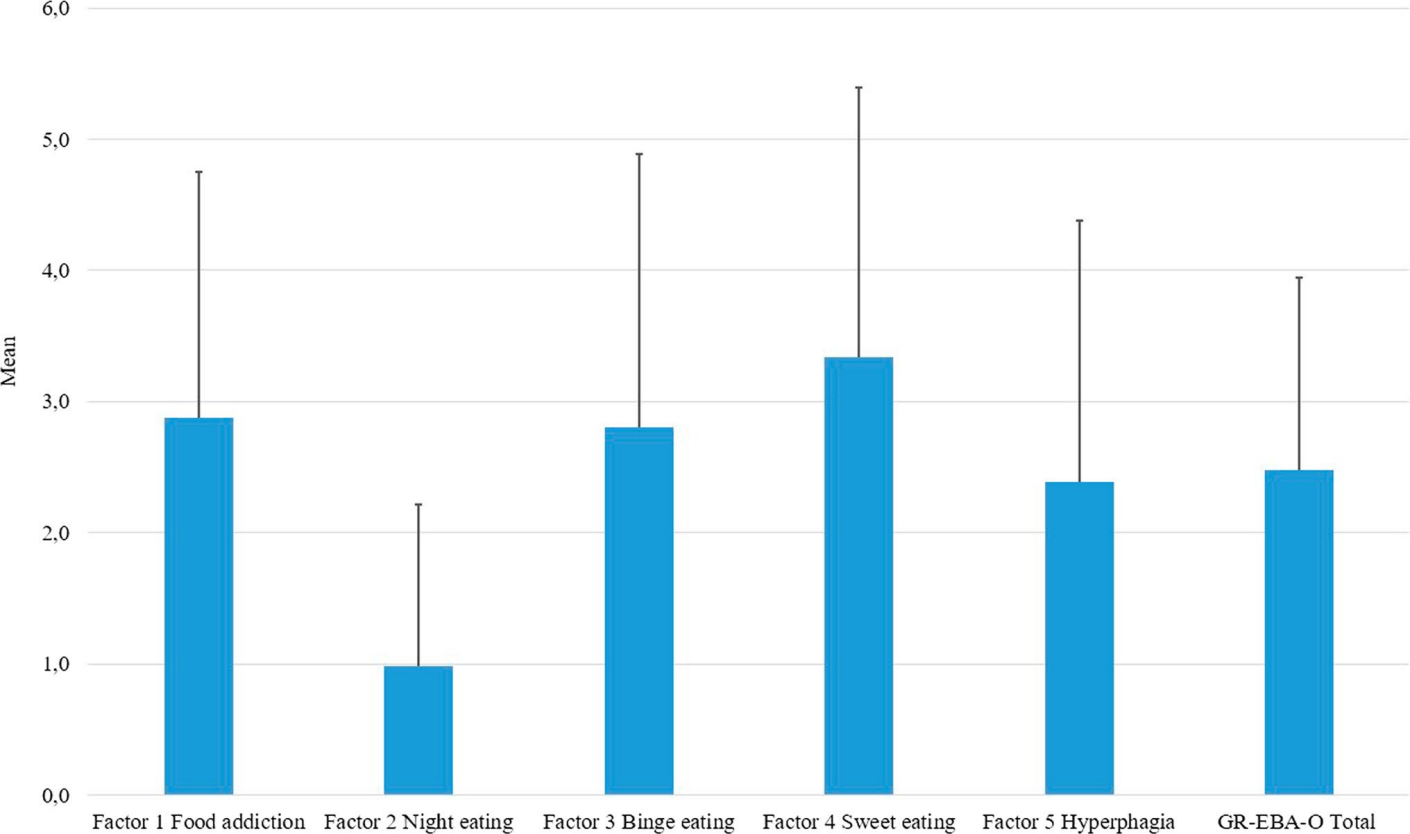
Means and standard deviation of GR-EBA-O factors and total score

Means and standard deviation of GR-EBA-O factors and total score in this study are displayed in Fig. 2.

## Discussion

The primary objective of this research was to validate the GR-EBA-O questionnaire. To our knowledge, no prior studies have explored the psychometric properties of the Greek version of this instrument. Regarding its structural composition, the original version suggested a second-order five-factor structure as the best fit. In our research, our findings align consistently with a second-order five-factor model, affirming that the total score effectively reassumes the characteristics of all the five factors.

Additionally, our reliability assessments yielded highly satisfactory results, displaying ω coefficients ranging from 0.87 to 0.92. These findings indicate commendable levels of omega reliability, valuable for clinical decisions. Segura-Garcia and colleagues also reported sound internal consistency in their results which ranged from 0.80 to 0.92 [20].

Regarding the convergent validity, significant positive correlations were found between the GR-EBA-O factors and the other psychopathological questionnaires in line with the findings of Segura-Garcia et al. [20], so the GR-EBA-O has proved to be appropriate to measure pathological eating behaviors.

Further, the investigation of eating behaviors indicated a good discriminant validity of the GR-EBA-O, in fact the MANOVA demonstrated that GR-EBA-O was a reliable instrument as no differences were found by sex and categorical BMI in the five factors of the scale, thus indicating the good reliability of the GR-EBA-O. Finally, in line with the original results [20], in this study we found the same score within the factors, in particular sweet eating subscale had the highest mean; while, night eating scored the lowest.

To date, there is no specific assessment available to intercept pathological eating behaviors associated with obesity. Employing the EBA-O as a self-assessment tool has proven beneficial in uncovering the intricate links between emotions and eating behaviors. Its user-friendly nature ensures accessibility even for those unversed in ED expertise. The robust psychometric properties of the EBA-O establish it as an easily applicable instrument in clinical settings, especially advantageous for professionals less familiar with EDs. In the pursuit of averting relapses into detrimental eating behaviors and drop-out treatment, screening for pathological eating behaviors serves as a valuable guide for physicians treating obesity, aiding in tailor-made interventions. Hence, leveraging the insights gleaned from the EBA-O results allows for the identification of patients at heightened risk of EDs, facilitating their referral to specialized ED units.

This study has some limits. Firstly, the data were obtained through self-reporting, potentially introducing biases. Secondly, the reliance on Internet-based data collection may have led to a sample of participants who self-selected [28]. Lastly, the absence of a re-test makes it challenging to determine the stability of the EBA-O as a measure of eating behavior but it is important to note that the EBA-O is intended as a state measure rather than a trait one.

## Conclusions

Summing up, the GR-EBA-O has demonstrated sound psychometric properties such as good model fit indexes and internal consistency. Thus, this study has shown that the Greek version of the EBA-o can be an easy-to-use and valid tool for clinicians and researchers in the self-report measurement of most present eating behaviors in patients with obesity.

## What is already known on this subject?

Pathological eating behaviors could contribute to identify different phenotypes of obesity. The EBA-O is a self-report measure developed to identify emotional-related eating behaviors in persons with obesity.

## What does this study add?

The Greek version of the EBA-O, GR-EBA-O, has been found to be a valuable, reliable and feasible tool to evaluate pathological eating behaviors for clinicians unskilled in the assessment of eating disorders.

## Data Availability

Data are available from the corresponding author upon request.
